# Dentistry and Dental Physicians

**Published:** 1875-10

**Authors:** Chas. H. Evans


### DENTISTRY AND DENTAL PHYSICIANS.

BY CHAS. H. EVANS.

Dentists have been represented by a writer in the Cosmos to be like a flock of sheep. If one of the sheep leaped over a wall all the others would follow; this reminds me of the following incident, it appears that lately a flock of twelve hundred sheep were passing along the edge of a precipice, the leader accidentally slipped off, and the whole twelve hundred jumped down after him and perished.

Now it would be singular if one person who thinks himself a leader in dentistry, should by a few flashy speeches induce a large number of dentists to set themselves up as dental physicians, by ignoring entirely the mechanical part of dentistry: because this formerly the principal part of the art, is at present over run by cheap bases and patents. This course in the present state of public opinion would only result in starvation to the majority of those who try it; for this reason, the public are not prepared for it, could not understand their position, and would not pay or support them.

At present, dentists, the world over, are looked on as but little more than skillful mechanics, and treated as such by the mass of the people. Some will say, educate the people! the people do not care to be informed in regard to dentistry. Even the plain common laws of health are fearfully disregarded, with the consequent loss of life. Why should we expect that dentistry should form an exception?

It will not do to let the mechanical part of dentistry slip out of the hands of the educated dentists; the intelligent portion of the public are looking to them for perfection in this department, and will continue to do so for many years to come. This has formed a part of the foundation on which the science and art of dentistry has been established and must continue to be in demand, for it forms the principal part of the dentistry required by the mass of the people.

If cheap bases for a time have cast a shadow over this part of our art, rather than it should be lost, let more thought and study be brought to bear on this work. Are all the treasures of knowledge exhausted ? has science nothing more to give ? is there nothing more to be learned? I believe there are magnificent results yet to be attained for the seeking, and the future will yet produce them in this part of dentistry.

It appears to me, that I can see a glimpse of this future, as applied to dentistry, by improvements and inventions in the arts similar to the following: a process has been recently perfected in which glass is rendered very tough and not easily broken; what could be neater than a gum colored, almost malleable glass plate. Take enough of such achievements as the above and the work is done.

We believe that in the not far distant future there will be produced by the dentists of course, a cement for filling teeth, enamel color and susceptible of a polish like healthy live enamel. It will require a most elaborate course of study and experiment to produce it; it must, and it will undoubtedly be produced.

It all rests with the dentists to decide what dentistry shall be, not one part, but each and every portion of it.

Some contend that dentistry requires so much science and skill that it should be divided. The uneducated public look on it as a trade, hardly that, easy, light, and genteel, and which most any dry goods clerk can learn in a few months, being a short road to riches (!) and gentility.

The demands of public opinion, although appearing at times somewhat "long-eared," cannot be be ignored with impunity at present; that is, if a person depends on the patronage of the mass of the people for support, or the good will of patients, of course no one desires the contrary.

I know an educated physician who advised a dentist to charge one dollar for advice and examination. If any one is of the opinion that he could get wealthy as a dental physician, let him charge this moderate fee, he will soon be able to form a correct opinion on this point.

Dentistry is none too large for it to receive the necessary respect which it should command. Elevate it so as to compel re spect for its attainments; the love of gain, trade and competition, have performed this service for porcelain teeth so that they are now very nearly perfect. Now, is it impossible that the plates for their support, may not be made their equal.

We are not unaware that there are now in use some approximations to this; there must be an adaptation to the wants and necessities of the public in every respect, for the work to command their appreciation. When this want is supplied its merits will create a demand for it.

Dentistry at present is somewhat in the condition of a besieged fortress, where one part is being stormed, by the demands of the mass of the people for cheap base, and the claims of those possessing the patents for the same. Now, the question is, shall this weak part be given up, or shall *men* enough be put in to hold it?

